# Efficacy of Siwan Traditional Therapy on Erythrocyte Sedimentation Rate, Lipid Profile, and Atherogenic Index as Cardiac Risk Factors Related to Rheumatoid Arthritis

**DOI:** 10.3390/medicina59010054

**Published:** 2022-12-27

**Authors:** Noha F. Mahmoud, Nashwa M. Allam, Islam I. Omara, Howida A. Fouda

**Affiliations:** 1 Rehabilitation Sciences Department, Health and Rehabilitation Sciences College, Princess Nourah bint Abdulrahman University, P.O. Box 84428, Riyadh 11671, Saudi Arabia; 2 Orthopedics and Orthopedic Surgery Department, Physical Therapy Faculty, Ahram Canadian University, Giza P.O. Box 12451, Egypt; 3 Department of Animal Production, Faculty of Agriculture, Cairo University, Giza P.O. Box 12613, Egypt; 4 Physical Therapy for Internal Diseases Department, Physical Therapy Faculty, October 6 University, Giza P.O. Box 12585, Egypt

**Keywords:** Siwa, Siwan traditional therapy, psammotherapy, cardiac risk factors, lipid profile, atherogenic index, physical function, rheumatoid arthritis

## Abstract

*Background and Objectives*: The most frequent cause of mortality in rheumatoid arthritis (RA) patients is cardiovascular disease (CVD). Inflammation, dyslipidemia, and decreased physical activity are some of the main risk factors for CVD. Siwan sand therapy is a type of traditional therapy used in Egypt to treat RA. The approach of this therapy depends on the experience of the healers. The aim of the current study was to compare the effects of three sessions of Siwan traditional therapy to five sessions on common CVD risk factors and physical function in rheumatoid arthritis patients. *Materials and Methods:* Thirty patients (9 male and 21 female) were assigned into two groups of equal size: group (A) received three sessions of Siwan traditional therapy in the form of a sand bath. Group (B) received the same form of therapy for five days. Erythrocyte sedimentation rate (ESR), lipid profile, atherogenic index of plasma (AIP), and a health assessment questionnaire (HAQ) were measured before and after treatment. *Results:* There was a significant increase above normal within group (A) for ESR (*p* = 0.001), triglycerides (TG; *p* = 0.015), total cholesterol (Tot-Chol; *p* = 0.0001), and low-density lipoprotein (LDL; *p* = 0.0001). However, there were no considerable differences in high-density lipoprotein (HDL; *p* = 0.106), very low-density lipoprotein (VLDL; *p* = 0.213), AIP (*p* = 0.648), and HAQ (*p* = 0.875). For the second group, there were significant changes within group B only in Tot-Chol (*p* = 0.0001), HDL (*p* = 0.0001), VLDL (*p* = 0.0001), AIP (*p* = 0.008), and HAQ (*p* = 0.014). There was a significant difference between both groups regarding HDL (*p* = 0.027), LDL (*p* = 0.005), AIP (*p* = 0.029), ESR (*p* = 0.016), and HAQ (*p* = 0.036). *Conclusions:* For RA patients, five days of Siwan traditional therapy caused significant changes regarding inflammation, Tot-Chol, LDL, HDL, AIP, and functional activity when compared to three days of Siwan hot sand therapy.

## 1. Introduction

Rheumatoid arthritis (RA) is a systemic, chronic, progressive autoimmune disease that primarily affects the linings of the joints (synovial membranes) [1,2]. The most affected populations with RA are women, smokers, and people with RA family history [3]. In developed countries, RA affects 0.5–1% of the adult population [1], with 40 new cases for every 100,000 people each year [4]. Considering that low-to-middle-income countries account for the vast majority of the world’s population, the number of affected people is significant and is expected to grow in the coming years [5].

Pathogenic pathways underpin the etiology of inflammatory rheumatic diseases, which are initiated by a systemic decrease in immunological tolerance and a subsequent disruption in the immune system. Multiple disorders outside of the joints are linked to systemic inflammation [6], such as vasculitis and uveitis, rheumatoid nodules, pericarditis, osteoporosis, rheumatoid lung [7], cardiovascular events, anemia, atherosclerosis, and type 2 diabetes mellitus [6].

Patients diagnosed with RA are 30–60% more likely to develop cardiovascular disease (CVD) than the general population [8]. In individuals with rheumatoid arthritis, the incidence of myocardial infarction and stroke was nearly doubled. Patients with rheumatoid arthritis exhibited a 30% increase in CVD mortality [9]. Approximately 30% of asymptomatic RA patients in a study group in upper Egypt had atherosclerosis compared to only 5% of normal control. Between the disease activity index and atherosclerosis, there was a strong statistically significant correlation [10]. CVD is a major mortality causing factor in RA patients [9]. The relationship between RA and CVD might be thought of as a “natural experiment” that, if properly analyzed, could shed light on the underlying processes by which inflammation speeds up the development of atherosclerosis, as well as heart disease [11]. Hence, the inflammation in RA is not confined to the joints but also present in the vessel wall. Previously believed to be a passive illness caused by lipid buildup, atherosclerosis is now widely understood to be a dynamic inflammatory process that starts with endothelial activation, leukocyte recruitment, and lipid oxidation and ends with plaque instability and thrombosis [12].

When compared to the general population, patients with RA have a 1.5–2.0-fold greater risk of developing coronary artery disease (CAD) [9], which is comparable to the risk posed by diabetes mellitus [13]. This elevated CAD risk is evident even before the clinical diagnosis of RA: before diagnosis, persons with RA were more than three times more likely to have had a previous MI than participants without RA. A European League Against Rheumatism expert group has advised that CV risk ratings in certain RA patients be multiplied by 1.5 to reflect their elevated risk of heart disease [14]. In individuals with RA, diastolic dysfunction may be linked to systemic inflammation, increasing the risk of heart failure [15].

In the general population, an unfavorable lipid profile, often called dyslipidemia, is a major risk factor for cardiovascular disease (CVD) [15,16]. Researchers found that the risk of cardiovascular disease steadily rose in correlation with blood cholesterol levels [17]. Convincing data suggests that people with rheumatoid arthritis (RA) have an increased risk of cardiovascular disease [9]. On the other hand, systemic inflammation appears to play a significant role in the lipid profile alterations seen in RA, making the relationship between lipids and cardiovascular risk in this disease more complicated than in the general population. Total cholesterol (Tot-Chol), low-density lipoprotein (LDL), and high-density lipoprotein (HDL) values are all lower in individuals with active, untreated RA, according to mounting data [18]. However, rising blood lipid levels may occur simultaneously with decreasing inflammation [19]. Uncertainty surrounds the effects of these modifications on cardiovascular risk, as well as the relative contributions of dyslipidemia and systemic autoimmune inflammation to this risk in RA [20].

Inflammatory indicators such as CRP, erythrocyte sedimentation rate, rheumatoid factor, anti-citrullinated protein antibodies, and more active or severe RA are all linked to an increased risk of cardiac disease in RA patients [21]. Even in people without rheumatic illnesses, rheumatoid factor and anti-nuclear antibodies are linked to heart disease and overall mortality [22]. Reduced muscle mass as well as an abnormal body mass index, which could be the result of uncontrolled inflammation, increase the likelihood that people with RA will engage in less physical activity [23]. HAQ, which measures functional activity, is a predictor of CVD and death [24]. The HAQ disability index (HAQ-DI) is a self-reported measurement of physical function. When evaluating the level of physical impairment caused by RA, the HAQ Disability Index (HAQ-DI) is the disability assessment component of the HAQ [25].

RA is a costly chronic systemic disease; therefore, it is important to explore different effective, cheap, and safe methods of therapy. Traditional sand therapy is one of the well-known traditional therapies in eastern countries, and Siwan sand therapy in Egypt is one such therapy. Siwan therapy is related to the word “Siwa”, which is an oasis in the western desert of Egypt. Siwan families have special skills in traditional healing. They pass their knowledge from generation to generation [26]. Siwan sand therapy was found to be more effective in improving functional activity and decreasing pain in RA patients than traditional physical therapy [27]. Generally, sand therapy can increase internal body temperature, decrease peripheral vascular resistance, increase tissue metabolism and venous return, and improve cardiac output, which in turn, increases the excretion of waste [28]. Despite the previously mentioned effects of sand therapy, it is not a popular intervention. Siwa Oasis has several traditional healing centers where sand therapy is applied. The centers receive patients from different governates in the country, most of them from rural areas or indigenous tribes on the borders of Egypt. Despite its great heritage value, there have not been enough studies conducted to validate its ideal approach and discover its underlying mechanism.

Per the authors’ knowledge, the current study is the first conducted to investigate the effect of Siwan sand therapy on some cardiac risk factors such as inflammation, lipid profile, atherogenic index, and physical function. Although cardiac events are the main cause of death in RA patients, the mechanism of their death is not well understood. Evidence suggests that people with RA are less likely to obtain either primary or secondary heart preventative medicine due to a lack of awareness among health providers and patients [15]. The current study aims to discover more about the effect of a cheap and safe treatment, sand therapy, on RA patients’ cardiac risk factors, considering that RA patients die mostly from CVD [9]. The study helps design the best practice protocol for such rare natural therapies.

## 2. Materials and Methods

### 2.1. Study Design

This study is a prospective single-blind pretest–posttest clinical trial. It was approved by the Ethics Committee of Cairo University’s Faculty of Physical Therapy, Egypt (P.T. REC/012/00947). The research adhered to the Declaration of Helsinki’s standards for the treatment of research participants. Research was conducted from March 2021 to August 2022. Siwan traditional therapy starts from June until the beginning of September annually.

### 2.2. Participants

Thirty RA patients, 9 males and 21 females, participated in this study according to the 2010 American College of Rheumatology/European League Against Rheumatism classification criteria for RA [29]. Patients were recruited from seven traditional healing centers in Siwa, Marsa Matrouh Governorate. They were interviewed and evaluated individually to assess their eligibility to be included in the study. Positive RA patients on a stable anti-rheumatic drug regimen; age range of 40 to 60 years; and BMI of 25 to 40 were the inclusion criteria. Patients who were known to have one of the following criteria were excluded from the study: uncontrolled diabetes, uncontrolled hypertension, renal disorders, unstable angina, heart failure, pregnancy, and bleeding disorders. Patients were assigned into two groups according to the number of treatment sessions.

### 2.3. Randomization

Patients were randomly assigned into two equal groups according to the number of treatment sessions. The authors did not interfere in selecting the number of sessions because it depended on the patient’s budget, tolerance, and preference after discussion with the traditional healers. However, a random list was generated within each group in an Excel sheet according to the patient order. Despite that, the principal healer knew the number of sessions for each group. The healer assistants who applied the process for the patients were blinded and did not know about the patients’ grouping till the end of the treatment day. All subjects gave their informed written permission before the initial assessment.

### 2.4. Intervention

Group (A) (*n* = 15) received Siwan sand therapy for three days, and Group (B) received Siwan sand therapy for five days. Sessions were conducted every day between 1 p.m. and 4 p.m. when the temperature of the sand was between 45 and 60 °C. It began with orientation the day before the sand baths began. Patients were advised not to take a shower or use body lotion or cream before the bath and not to use a fan or air conditioning during the sand bathing period and for three days after treatment. The patients were instructed to cover their bodies well to prevent air drafts and drink a significant amount of hot or warm fluids before and after every session to prevent dehydration. In the early morning of the next day, healers traveled to El Dakrour Mountain to dig holes in the sand. The hot radiation of the sun increases the sand temperature until noon. The patients were asked to lay supine in the hole. Then, healers covered their bodies with sand, except for the neck and head regions, which were kept under a small umbrella. The sand bath continued for 15–30 min as tolerated by the patient, then they were well wrapped and transferred to a dry tent close to the hole. The patients were seated inside the tent for 10–15 min and were given herbal warm drinks such as anise and lemon juice. Sweating and a number of physiological changes take place for two hours from the beginning of the session. After the sweating stopped, patients were able to change clothes into dry and heavy ones and were allowed to go to the hotel for rest. After three and five sessions of sand bathing, groups (A) and (B) received a whole-body massage with olive oil. Patients rested until the morning and were then allowed to travel back home.

### 2.5. Outcome Measures

#### 2.5.1. Lipid Profile

Composed of Tot-Chol, TG, HDL, LDL, and VLDL were assessed according to standard laboratory protocol [30]. Interpretations of the lipid profile:

- Tot-Chol: normal, up to 200 mg/dL; borderline, 200–239 mg/dL; high, more than 240 mg/dL.
- TG: normal female, 35–135 mg/dL; normal male, 40–160 mg/dL.
- HDL: 45–65 mg/dL is considered normal.
- LDL: normal, less than 100–130 mg/dL; borderline high, 130–159 mg/dL; high, 160–189 mg/dL.
- VLDL: normal, 25–50 mg/dL.

#### 2.5.2. Atherogenic Index of Plasma (AIP)

Measured by (AIP = log10 TG/HDL) [31,32].

- Lower values are associated with a lower risk of cardiovascular disease.
- Values between 0.11 and 0.21 are associated with intermediate risks.
- High risks are associated with values greater than 0.21.

#### 2.5.3. Inflammatory Marker

As a common and cheap hematology test, the erythrocyte sedimentation rate (ESR) was used to indicate and monitor the increased inflammatory activity, according to the International Council for Standardization in Hematology (ICSH) [33]. The ESR fast detector machine was used for the test. Its normal value is up to 7 mm/s.

#### 2.5.4. Physical Function

The HAQ disability index examines a patient’s functional capacity and includes questions on fine upper-extremity movements, locomotor activities of the lower limb, and tasks that include both the upper and lower extremities. Twenty questions are broken down into eight different categories of functioning, each of which represents a full set of functional tasks. These categories include clothing, getting up, eating, walking, hygiene, reaching, and gripping, as well as daily activities. Each question begins with the phrase “Are you able to...?” before moving on to the specific activity. On a scale from 0 (no disability) to 3 (severe impairment) (completely disabled), the patient’s answers are written down. At least two distinct component questions are included in each of the categories [34]. The HAQ-Arabic version was employed in the present investigation [35].

- 0 to 1, 0 indicates mild to moderate difficulty
- 1 to 2 indicates moderate to severe difficulty
- 2 to 3 indicates severe to very severe difficulty.

### 2.6. Sample Size Effect

The appropriate sample size for this study is 30 patients (15 patients in each group). The G*Power software version 3.1.9 (G*Power program version 3.1, Heinrich-Heine-University, Düsseldorf, Germany) was used to calculate the two-tailed test sample size. The sample size calculation was dependent on t-tests (means: difference between two independent means for two groups), type I error (alpha = 0.05), power (1-βeta = 90%), and the effect size d = 0.95.

## 3. Results

### 3.1. Statistical Analysis

In the current study, data were normally distributed after using the Shapiro–Wilk test (*p* > 0.05) and Levene’s test for testing the homogeneity of variance (*p* > 0.05). SPSS Package, version 25 for Windows, was used to conduct the statistical analysis (SPSS, Inc., Chicago, IL, USA). Statistical measures for continuous data are the mean and standard deviation, whereas those for discrete data are numbered categories (percentage). For numerical data, a paired *t*-test was utilized to compare the two groups pre- and post-treatment, and an unpaired *t*-test was utilized to compare the two groups pre- and post-treatment. For categorical data, the Chi-square test was utilized for within-group, between-group, and subgroup comparisons. When the level of probability is less than or equal to 0.05 (*p* ≤ 0.05), the data is considered significant.

### 3.2. Results

An overall number of 30 rheumatoid patients from both genders (9 males and 21 females) were involved in this study and randomized into two groups (15 patients per group). The statistical analysis for demographic data (Table 1) revealed that there were no significant differences (*p* > 0.05) in age (*p* = 0.617), BMI (*p* = 0.834), disease duration (*p* = 0.407), gender (*p* = 0.690), diabetes (*p* = 0.624), hypertension (*p* = 0.409), heart problems (*p* = 0.143), poor lipids (*p* = 0.232), medication (*p* = 0.464), academic level (*p* = 0.295), and history of previous Siwan Traditional Therapy (*p* = 0.705) between the two experimental groups.

The distribution of lipid profiles, AIP, and ESR in (Table 2) revealed that there were significant differences between before and after treatment for triglycerides (*p* = 0.015), Tot-Chol (*p* = 0.0001), LDL (*p* = 0.0001), and 1st hour ESR (*p* = 0.001) in group (A) pairwise comparison tests. However, there were no significant differences in HDL (*p* = 0.106), VLDL (*p* = 0.213), and AIP (*p* = 0.648) within the 3-day group. In group (B) (Table 2), pairwise comparison testing showed that there were significant differences between pre- and post-treatment for Tot-Chol (*p* = 0.0001), HDL (*p* = 0.0001), VLDL (*p* = 0.0001), and AIP (*p* = 0.008) but no significant differences in TG (*p* = 0.406), LDL (*p* = 0.580), and 1st hour ESR (*p* = 0.878) within the 5-day group. Group (B) recorded fewer changes in all laboratory investigations (TG, Tot-Chol, HDL, LDL, VLDL, and 1st hour ESR) (5.63, 29.76, 3.98, 3.10, 3.88, and 1.04, respectively) compared to group (A) (31.37, 35.25, 6.76, 31.01, 5.79, and 28.17, respectively). At pre-treatment (Table 2), pairwise comparison tests (group effect) revealed no significant differences in all studied laboratory investigations. After treatment (Table 2), HDL (*p* = 0.027), LDL (*p* = 0.005), AIP (*p* = 0.029), and 1st ESR (*p* = 0.016) were affected significantly (*p* < 0.05) between both groups. However, there were no substantial differences among the 3-day group and the 5-day group in Siwan traditional therapy, TG (*p* = 0.340), (*p* = 0.559), VLDL (*p* = 0.503). A further subgroup analysis is seen in Table 3.

Table 3 shows the comparison of lipid profiles and AIP in subgroups; there was a significant (*p* = 0.041) increase in normal HDL values after Siwan Traditional Therapy and a non-significant increase in normal TG (*p* = 0.439), LDL (*p* = 0.796), and VLDL (*p* = 0.670) values in group (B).In group A, there was a non-significant increase in normal values of VLDL (*p* = 0.593) and a non-significant decrease in normal values of TG (*p* = 0.835) and HDL (*p* = 0.257), but there was no change in normal values of LDL (*p* = 1.000). Moreover, no change was noted in total cholesterol profiles in both groups. In group B, there was a significant decrease in high AIP risk (*p* = 0.035) and a non-significant increase in moderate risk (*p* = 0.593) while increasing the percentage of low risk. However, in group A, there was a non-significant decrease in high risk (*p* = 0.655), a non-significant increase in moderate risk (*p* = 0.414), and no change in low risk (*p* = 1.000). In general, there were significant improvements in HDL and AIP and non-significant improvements in TG, LDL, and VLDL due to the treatment of rheumatoid patients with 5-day traditional sand therapy (Group B) compared with 3-day traditional sand therapy (Group A).

The distributions of HAQ (Table 4) did not differ significantly between before and after treatment within the 3-day group (*p* = 0.875), but there were substantial differences in HAQ within the 5-day group (*p* = 0.014). There were no significant differences in HAQ before treatment (*p* = 0.656), but there were substantial differences in HAQ at post-treatment (*p* = 0.036) between Groups (A) and (B).

## 4. Discussion

Sand therapy has been used for centuries to improve function and reduce pain in patients [36]. Siwa is a place that embraces that practice. However, modern science has not investigated its benefits and hazards extensively. Therefore, the current study evaluated the efficacy of Siwan traditional sand therapy on some cardiac risk factors. It is a trial to uncover few physiological changes that happen to RA patients because of that approach, which is directed mainly toward pain and function rather than its mechanism or other effects. In a trial to standardize this method, the study also compares the effects of 3 days versus 5 days of treatment, as traditional healers usually, from their experience, recommend an odd number of sessions: three, five, or seven.

The main risk factors for CVD in the overall population include dyslipidemia, hypertension, obesity, lack of physical activity, poor nutrition, and smoking [37]. Disease activity scores and inflammation are important risk factors for CVD in RA patients as there is a significant association between them [38]. Therefore, controlling inflammation is important to reduce CV events [39]. Inflammation was assessed using the ESR test. An elevated ESR is an important detector of coronary artery disease [40].

The results of the current study revealed that in the 3-day group, there was a significant increase when comparing before and after treatment for ESR (*p* = 0.001), while there was an insignificant decrease in the second group (*p* > 0.05), showing that ESR (a measure of inflammation) went up in the 3-day group and went down in the 5-day group. The increase in inflammatory biomarkers (ESR) in the 3-day group may be due to the immediate effect of generalized hyperthermia. The patient is exposed to a high temperature (from 45 to 60 °C) for 15 to 30 min, which might have stimulated thermo-nociceptors called Transient Receptor Potential Vanilloid 1 (TRPV1), which in turn, can increase inflammation by the production of interleukin-6, interleukin-8, and prostaglandin, thereby reducing inflammation [41]. Thermal therapy might reduce inflammation and repair cartilage damage by preventing the binding of serum tumor necrosis factor-a (TNF) to activated cells that produce pro-inflammatory cytokines [42].

The Siwan sand mineral constituents may explain the beneficial effect of the current study when augmented by the effect of hot sand. The hot sand bath increases skin permeability, which might aid the transport of the sand’s mineral constituents to the skin’s deeper layers, allowing them to do their work [43]. Siwan sand was analyzed in a previous study in 2018, and it was found to be rich in carbon, silicon, Ca, and Mg, plus other microelements [27]. The elements Ca (in ionic form) and Zn (in covalent form) can enter the dermis and be absorbed by skin cells. Ca^2+^ plays an important role in maintaining healthy muscle and nervous system function, as well as regular cardiovascular function. Calcium treatment in conjunction with vitamin D treatment is also known to improve calcium absorption. As a result, sand treatment is beneficial to human health and may have a role in reducing musculoskeletal disease and enhancing functional impairment. Sand walking is a great way to exercise and obtain vitamin D from the sun [44], which in turn, might have affected the results of HAQ.

Low magnesium (Mg) levels are linked to increased inflammation [45]. For example, magnesium salts in Dead Sea water have a beneficial effect on inflammatory diseases [46]. Moreover, in 1966, the German chemist Bedouno Sanouni analyzed the sands near Siwa. Radon levels were reportedly greater than in neighboring areas. Geological research revealed silica carbonates, iron, and magnesium in quite high concentrations. Radon therapy is recommended for rheumatoid arthritis and has been used in rheumatoid arthritis treatment since the beginning of the 20th century [47]. Radon is taken up by transcutaneous resorption, which may be facilitated by carbon dioxide or heat [48], and then it is distributed all over the body by the blood. Radon stimulates the release of anti-inflammatory cytokines. These cytokines act as antagonists against the pro-inflammatory cytokines [49].

Results of the lipid profile of the current study showed that in Group (A), there was no significant increase in the normal value before and after treatment for VLDL, while there was no significant increase in the abnormal values of TG, Tot-Chol, HDL, and LDL. In Group (B), there was a significant increase in normal values of HDL pre- and post-treatment but no significant increase in normal values of VLDL pre- and post-treatment.

The plasma lipid profile is a major risk factor and predictor of CVD [50]. A strong association was found between low values of HDL, high levels of LDL, and cardiovascular events [51]. Chronic inflammation in RA leads to quantitative as well as qualitative changes in TG, LDL, and HDL [52]. It leads to a condition of “reverse epidemiology”. Patients in remission from their RA no longer have the “lipid paradox” of high CV risk among those with low LDL cholesterol [53]. Therefore, the increased inflammation in the 3-day group in the current study might explain the increased number of patients with abnormal values of TG, Tot-Chol, HDL, and LDL. Group (B) results are aligned with a study conducted on healthy young males who received 10 hot sauna sessions. LDL and cholesterol fraction levels dropped throughout the sauna sessions. However, after sauna sessions, some people noticed a small (but not statistically significant) increase in HDL and a temporary decrease in TG [54]. The present study contradicted a study that showed a statistically significant rise in blood Tot-Chol with small as well as statistically insignificant shifts in LDL and HDL fractions in a group of middle-aged patients subjected to a set of 20-min bathing sessions in natural hot springs with temperatures of 42 °C two times a week for three months [55]. The difference between this result and the current study result may be due to differences in both temperature and/or treatment approach.

One factor that may explain the tendency of the lipid profile toward normal values in the 5-day group is the presence of magnesium, which is very important for human health. Under the form of Mg+2, magnesium produces well-known effects based on animal experimentation “in vivo”, reducing cardiovascular pathologies since it has an important role in the metabolism of fats or lipids [44]. Although both groups took the same sessions at different doses, the results were different. It might be recommended that patients suffering from dyslipidemia need higher doses of thermal and magnesium therapy. The impact of lipid fluctuation due to the variable grade of chronic inflammation on CV risk is less well understood throughout the disease process [56]. The fluctuations in increased inflammation in the 3-day group and a decrease in the 5-day group might have affected the lipid profile results and made them even more difficult to explain, especially with the known lipid paradox in RA patients. Therefore, the AIP may be a better choice for assessing the relative impact of lipids on CV risk in those patients than the specific cholesterol fraction tests [44,56].

The atherogenic index of plasma (AIP) is a powerful predictor of atherosclerosis, as well as coronary heart disease. It reflects the genuine link between protective and atherogenic lipoproteins and is related to the particle size of pre- and anti-atherosclerotic lipoproteins. It may be determined using the log (TG/HDL-C) equation [51]. The results showed that there was a non-significant change in Group A and a significant change in the second group. A significant difference was also found between groups. The non-significant decrease in AIP in Group (A) might be related to the increased inflammation in that group, which affected the results of TG and HDL, while the significant improvement in AIP in Group (B) might be related to the decreased inflammation and its effect on TG and HDL. ESR is positively correlated with AIP in RA patients and is a predictor of AIP. Rheumatoid arthritis is linked to an altered lipid profile, particularly in individuals with elevated inflammatory markers, as well as autoimmune antibodies [57].

Fatigue, anemia, and muscle wasting accompany chronic inflammation with other specific disease symptoms, leading to deconditioned muscles. Comorbidities exacerbate inflammation, which in turn, negatively affects physical activity and cardiovascular performance. This is the “vicious cycle” of chronic inflammation underlying inflammatory rheumatic disorders [58]. Physical inactivity is one of the risk factors for cardiac events in RA patients, and these results can be explained by the fact that inflammation was lower in group B than in group A. The results might also be attributed to the physiological effects of sand therapy, as hot sand baths can significantly increase body temperature, which in turn, improves muscle tonicity and decreases pain [34]. It also reduces peripheral arterial resistance and increases blood flow [59], which in turn, increases tissue metabolism and oxygenation [60]. These results are in agreement with a previous study where seven sessions of Siwan sand therapy improved functional disability in RA patients [27]. However, it came into conflict with the short-term measures of another previous study [61], which found that seven sessions of Siwan sand therapy on RA patients decreased functional activity. Both studies were conducted in Siwa, but the measurement was performed after different treatment durations and/or treatment protocols from the current study.

Increasing physical activity could lead to better disease control, as recommended by recent international guidelines, and thus, improve the CV profile. Physical function measured by HAQ could be an indicator of physical activity, as HAQ is significantly associated with physical activity [34]. Physical function measured by HAQ in the first group improved but not significantly. It has, however, significantly improved in group B, as well as between the two groups.

These results can be explained by the fact that inflammation was lower in group B compared to group A. The results might also be attributed to the physiological effects of sand therapy, as hot sand baths can significantly increase body temperature, which in turn, improves muscle tonicity and decreases pain [34]. It also reduces peripheral arterial resistance and increases blood flow [59], which in turn, increases tissue metabolism and oxygenation [60]. These results are in agreement with a previous study where seven sessions of Siwan sand therapy improved functional disability in RA patients [27]. However, it came in conflict with the short-term measures of another previous study [61], which found that seven sessions of Siwan sand therapy on RA patients decreased functional activity. Both studies were conducted in Siwa, but the measurement was performed after different treatment durations and/or treatment protocols different from the current study.

Hippocrates, known as the “Father of Natural Medicine,” thought that because man is part of the Cosmos, nature may heal him. He viewed health as the perfect condition of equilibrium among natural forces, and he felt that physicians should consider the curative power of vital energy. He suggested sunbathing, water, and detoxifying to attain this purpose [44]. It is worth mentioning that there is something else beyond the effect of hot sands that might have boosted the results of this study, which is the harmony between natural forces that is found in Siwa. Maybe it is the special location, below sea level by up to 18 m, the very dry and hot weather, the ecological architecture, the mineral springs, and the salt lakes that work together to encourage body self-healing [36] in a way that is known as climatotherapy rather than sand therapy.

## 5. Conclusions, Limitations, and Recommendation

The study found that 5-days Siwan hot sand therapy caused significant changes regarding inflammation, Tot-Chol, LDL, HDL, AIP, and functional activity when compared to 3-days of Siwan hot sand therapy. Five sessions of Siwan sand therapy might be able to reduce cardiovascular risk factors more than three sessions of therapy. More investigations are needed to explore the underlying mechanisms of such measured effects. The clinical implementation of the current results still needs further research and comparison between RA patients, a normal subject group, and a RA placebo group. Further studies are needed to evaluate the chronic effects of longer sand therapy sessions and to establish a standardized sand bath therapy protocol for the various clinical conditions and different approaches to setting an effective treatment session.

The authors considered that the measured biological variables could be changed because of the climate of the place (climatotherapy) rather than only sand therapy. That is why the control group should be in Siwa. However, the current study is limited to the two experimental groups without a control group, which was not convenient for the patients (due to the long distance and the fatigability of the disease, as well as for the research budget). It was also considered unethical to transport patients over long distances without providing treatment. Further randomized control trials with a larger sample size are recommended for future research. The research is limited to 30 patients. However, this type of intervention is only available 70 days a year and for a limited number of patients.

It is also important to highlight that this study aimed to investigate the effect of Siwan sand therapy, which is a non-invasive, known, and widely used approach by RA patients. It did not aim to assess its effectiveness. Rheumatoid patients usually target symptomatic relief rather than the risk factors. The current study measured inflammation, lipid profile, atherogenic index, and physical function as a trial to give a deep look inside the physiological changes due to this kind of therapy. More research is needed to extensively explore the underlying effects of such interventions. Cardiac risk factors were selected as the leading cause of death among RA patients.

## Figures and Tables

**Table 1:** Rheumatoid patient’s general characteristics in 3-day group and 5-day group.

Items	Groups		*p*-Value
	Group A (*n* = 15) 3-Day Group	Group B (*n* = 15) 5-Day Group	
Age (year)	48.89 ± 8.38	49.80 ± 9.34	0.617
BMI (kg/m^2^)	30.83 ± 6.42	32.37 ± 5.77	0.834
Disease duration (year)	10.40 ± 2.71	11.11 ± 3.29	0.407
Gender (Males:Females)	5 (33.3%):10 (66.7%)	4 (26.7%):11 (73.3%)	0.690
Diabetes (Yes:No)	2 (13.3%):13 (86.7%)	3 (20.0%):12 (80.0%)	0.624
Hypertension (Yes:No)	3 (20.0%):12 (80.0%)	5 (33.3%):10 (66.7%)	0.409
Heart problem (Yes:No)	0 (0%):15 (100%)	2 (13.3%):13 (86.7%)	0.143
Poor lipids (Yes:No)	6 (40.0%):9 (60.0%)	3 (20.0%):12 (80.0%)	0.232
Medication (Medicated:Non-medicated)	7 (%):8 (53.3%)	9 (60.0%):6 (40.0%)	0.464
Academic level (Educated:Non-educated)	13 (86.7%):2 (13.3%)	10 (66.7%):5 (33.3%)	0.195
Have you ever been through STT before (Yes:No)	6 (40%):9 (60%)	5 (33.3%):10 (66.7%)	0.705

Unpaired *t*-test is used to compare numerical data expressed as mean ± standard deviation. Chi-square test is used to compare categorical data expressed as numbers (percentage). *p*-value: probability value; NS: non-significant; STT: Siwan Traditional Therapy.

**Table 2:** Inter- and intra-groups comparison for laboratory investigations.

Items		Groups (Mean ± SD)		Change (MD)	*p*-Value
		Group (A) 3-Days (*n* = 15)	Group (B) 5-Days (*n* = 15)		
TG	Before-treatment	160.00 ± 23.89	161.30 ± 61.99	1.30	0.968
	After-treatment	128.63 ± 92.08	155.67 ± 66.58	27.04	0.340
	Change (MD)	31.37	5.63		
	Change %	19.60%	3.49%		
	*p*-value	0.015 *	0.406		
Tot-Chol	Before-treatment	161.62 ± 48.29	160.90 ± 35.63	0.72	0.959
	After-treatment	196.87 ± 35.98	190.67 ± 24.80	6.20	0.559
	Change (MD)	35.25	29.76		
	Change %	21.81%	18.60%		
	*p*-value	0.0001 *	0.0001 *		
HDL	Before-treatment	48.87 ± 18.06	46.10 ± 9.79	2.77	0.560
	After-treatment	55.63 ± 22.77	42.11 ± 9.01	13.52	0.027 *
	Change (MD)	6.76	3.98		
	Change %	13.83%	8.63%		
	*p*-value	0.106	0.002 *		
LDL	Before-treatment	146.43 ± 23.52	142.54 ± 42.22	3.89	0.744
	After-treatment	115.42 ± 24.15	139.44 ± 21.98	24.02	0.005 *
	Change (MD)	31.01	3.10		
	Change %	21.17%	2.17%		
	*p*-value	0.0001 *	0.580		
VLDL	Before-treatment	30.45 ± 29.88	32.16 ± 12.40	1.71	0.817
	After-treatment	24.66 ± 19.51	28.28 ± 10.90	3.62	0.503
	Change (MD)	5.79	3.88		
	Change %	19.01%	12.06%		
	*p*-value	0.213	0.0001 *		
AIP Before treatment	Low risk (<0.11) Moderate risk (0.11–0.21) High risk (>0.21)	2 (13.3%)	0 (0.0%)		
		2 (13.3%)	6 (40.0%)		0.122
		11 (73.3%)	9 (60.0%)		
AIP after treatment	Low risk (<0.11) Moderate risk (0.11–0.21) High risk (>0.21) *p*-value	2 (13.3%)	5 (33.3%)		
		4 (26.7%)	8 (53.4%)		0.029 *
		9 (60.00%)	2 (13.3%)		
		0.648	0.008 *		
ESR (1^st^ hour)	Before-treatment	41.53 ± 13.98	44.33 ± 28.86	2.80	0.715
	After-treatment	69.70 ± 3 2.44	43.29 ± 26.50	25.41	0.016 *
	Change (MD)	28.17	1.04		
	Change %	67.83%	2.35%		
	*p*-value	0.001 *	0.878		

Data are expressed as mean ± standard deviation (SD) for TG, Tot-Chol, HDL, LDL, VLDL, and ESR. Data are expressed as number percentage for atherogenic index. MD: mean difference, *p*-value: probability value, * Significant (*p* < 0.05).

**Table 3:** Subgroup comparison of lipid profiles.

Lipid Profiles	Categories	Group A (3-Days) (*n* = 15)		*p*-Value	Group B (5-Days) (*n* = 15)		*p*-Value
		Before-Treatment	After-Treatment		Before-Treatment	After-Treatment	
TG	Low	0%	0%	-	0%	0%	-
	Normal	83%	75%	0.835	42%	58%	0.439
	High	17%	25%	0.705	58%	42%	0.439
Tot-Chol	Low	100%	75%	0.433	92%	67%	0.414
	Normal	0%	0%	-	0%	0%	-
	Borderline	0%	17%	-	0%	33%	-
	High	0%	8%	-	8%	0%	-
HDL	Low	44%	55.3%	0.796	66.6%	46.66%	0.046 *
	Normal	33.33%	11.36%	0.257	33.3%	53.33%	0.041 *
	High	22.6%	33.33%	0.480	0%	0%	-
LDL	Low	33.33%	11.36%	0.257	11.11%	0%	-
	Normal	55.31%	55.31%	1.000	44.44%	53.33%	0.796
	Borderline	0%	11.11%	-	33.33%	40.00%	0.763
	High	11.36%	22.22%	0.655	11.11%	6.67%	0.564
VLDL	Low	42%	38%	0.989	25%	17%	0.705
	Normal	42%	54%	0.593	67%	83%	0.670
	High	17%	8%	0.317	8%	0%	-
AIP	Low risk	13.30%	13.30%	1.000	0.00%	33.30%	-
	Moderate risk	13.30%	26.70%	0.414	40.00%	53.40%	0.593
	High risk	73.30%	60.00%	0.655	60.00%	13.30%	0.035 *

Data are expressed as number percentage *p*-value: probability value * Significant (*p* < 0.05).

**Table 4:** Inter- and intra-groups comparison for HAQ.

Variables		Categories	Groups		*p*-Value
			Group (A) 3-Days Group (*n* = 15)	Group (B) 5-Days Group (*n* = 15)	
HAQ	Before- treatment	Mild—moderate difficulty (0–1)	5 (33.3%)	4 (26.70%)	0.656
		Moderate—severe disability (1–2)	8 (53.3%)	7 (46.7%)	
		Severe—very severe disability (2–3)	2 (13.3%)	4 (26.7%)	
	After- treatment	Mild—moderate difficulty (0–1)	5 (33.3%)	12 (80.0%)	0.036 *
		Moderate—severe disability (1–2)	7 (46.7%)	2 (13.3%)	
		Severe—very severe disability (2–3)	3 (20.0%)	1 (6.7%)	
	*p*-value		0.875	0.014 *	

Data are expressed as number (percentage). *p*-value: probability value * Significant (*p* < 0.05).

## Data Availability

We declare that the data considered for this research are original and were collected by the authors for gaining insights.
